# Supplementary material for: Splice-Junction-Based Mapping of Alternative Isoforms in the Human Proteome
Source: Cell Rep. Author manuscript; Available in PMC 2020 Jan 15. (PMC6961840; doi:10.1016/j.celrep.2019.11.026)
Supplement: 2 [file NIHMS1546469-supplement-2.zip › DF1/PXD010154/Liver/Liver_45_MPST_AGVGAMGGGGAAGPARWAASAAAGR.pdf]

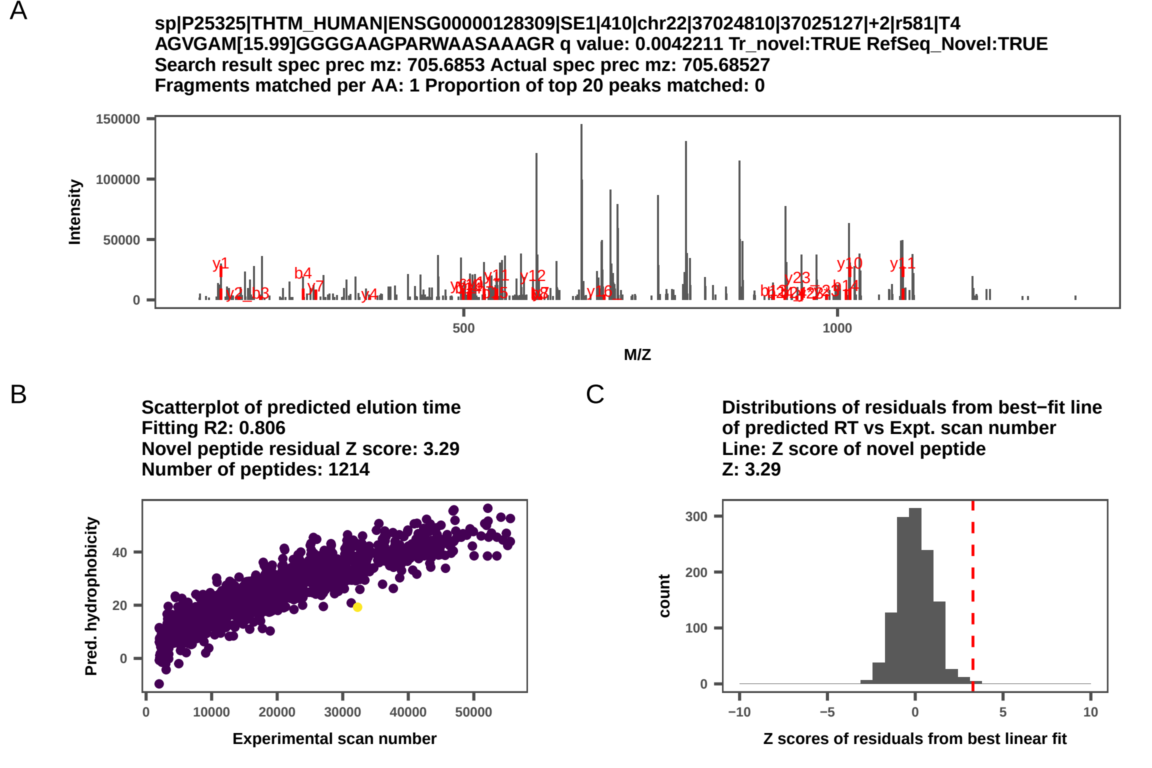
A
sp|P25325|THTM_HUMAN|ENSG00000128309|SE1|410|chr22|37024810|37025127|+2|r581|T4
AGVGAM[15.99]GGGGAAGPARWAASAAAGR q value: 0.0042211 Tr_novel:TRUE RefSeq_Novel:TRUE
Search result spec prec mz: 705.6853 Actual spec prec mz: 705.68527
Fragments matched per AA: 1 Proportion of top 20 peaks matched: 0
Intensity
150000
100000
50000
0
500
1000
M/Z
y1
b4
y7
y4
y11
y12
y16
y23
y10
y11
B
Scatterplot of predicted elution time
Fitting R2: 0.806
Novel peptide residual Z score: 3.29
Number of peptides: 1214
Pred. hydrophobicity
40
20
0
0
10000
20000
30000
40000
50000
Experimental scan number
C
Distributions of residuals from best−fit line
of predicted RT vs Expt. scan number
Line: Z score of novel peptide
Z: 3.29
count
300
200
100
0
−10
−5
0
5
10
Z scores of residuals from best linear fit
